# Prevalence and Prognostic Value of Right Ventricular–Pulmonary Artery Uncoupling in Adults with Right-Sided Congenital Heart Disease

**DOI:** 10.3390/jpm16030164

**Published:** 2026-03-16

**Authors:** Giulia Iannaccone, Alessandro Olimpieri, Maria C. Meucci, Rosa Lillo, Maria Grandinetti, Alessio Cianci, Claudio Di Brango, Angelica B. Delogu, Massimo Massetti, Gaetano A. Lanza, Antonella Lombardo, Francesca Graziani, Francesco Burzotta

**Affiliations:** 1 Department of Cardiovascular Sciences-CUORE, Fondazione Policlinico Universitario A. Gemelli IRCCS, 00168 Rome, Italy; giulia.iannaccone@guest.policlinicogemelli.it (G.I.); mariachiara.meucci@guest.policlinicogemelli.it (M.C.M.); rosa.lillo@policlinicogemelli.it (R.L.); maria.grandinetti@policlinicogemelli.it (M.G.); angelicabibiana.delogu@policlinicogemelli.it (A.B.D.); massimo.massetti@policlinicogemelli.it (M.M.); gaetanoantonio.lanza@policlinicogemelli.it (G.A.L.); antonella.lombardo@policlinicogemelli.it (A.L.); francesco.burzotta@policlinicogemelli.it (F.B.); 2 Department of Cardiovascular and Pulmonary Sciences, Catholic University of the Sacred Heart, 00168 Rome, Italy; alessandro.olimpieri@guest.policlinicogemelli.it (A.O.); alessiocianci.1998@gmail.com (A.C.); claudio.dibrango@guest.policlinicogemelli.it (C.D.B.)

**Keywords:** adult congenital heart disease, right ventricular–pulmonary artery uncoupling, TAPSE/PASP, right-sided CHD, prognosis, need for intervention

## Abstract

**Background:** Right-sided congenital heart diseases (R-CHDs) are frequently associated with right ventricular (RV) dysfunction and heterogeneous clinical trajectories, underscoring the need for individualized risk assessment. RV–pulmonary artery (RV–PA) coupling has emerged as an important prognostic marker in several cardiovascular conditions. However, its role in adults with R-CHD has not been well established. **Methods:** We retrospectively reviewed consecutive adults with R-CHD evaluated at our outpatient clinic between October 2013 and November 2023. RV–PA uncoupling was defined by echocardiography as a tricuspid annular plane systolic excursion (TAPSE) to pulmonary artery systolic pressure (PASP) ratio ≤0.55. The primary composite endpoint included all-cause mortality, major supraventricular and ventricular arrhythmias, unplanned cardiac hospitalizations, and need for (re)-interventions. **Results:** A total of 132 patients (mean age 41.6 ± 15.7 years; 51.5% male) were included. RV–PA uncoupling was identified in 48 patients (36.4%). Over a median follow-up of 40.3 months, the primary composite endpoint occurred in 71 patients (53.8%). Patients experiencing adverse outcomes were older and showed lower TAPSE, higher PASP, larger RV and right atrial dimensions, and a significantly higher prevalence of RV–PA uncoupling (*p* < 0.001). Multivariable Cox regression analysis demonstrated that RV–PA uncoupling was a strong independent predictor of adverse outcomes (HR 4.478, *p* < 0.001), outperforming its individual components. In addition, RV–PA uncoupling and RV mid-diameter independently predicted the need for surgical or interventional procedures during follow-up. **Conclusions:** RV–PA uncoupling provides robust and independent prognostic information in adults with R-CHD and represents a practical tool for personalized risk stratification, potentially guiding tailored surveillance strategies and timing of therapeutic interventions.

## 1. Introduction

In patients with right-sided congenital heart disease (R-CHD), progressive right ventricular (RV) dysfunction and pulmonary vascular hypertension may develop over time due to volume overload, pressure overload, or both [1]. Recently, the ability of the RV to face a given pulmonary artery (PA) afterload—referred to as RV-PA coupling—has been identified as a valuable predictor of clinical outcomes across a wide range of clinical settings [2,3,4,5,6]. Echocardiography offers the opportunity to assess RV-PA coupling non-invasively and in a validated manner, through the calculation of the ratio of tricuspid annular plane systolic excursion (TAPSE) to pulmonary artery systolic pressure (PASP) [7]. The TAPSE/PASP ratio has emerged as a prognostic marker in various conditions, including heart failure [2,8], valvular heart disease [4,9,10], and pulmonary arterial hypertension [3,11]. Furthermore, the use of the TAPSE/PASP ratio with a cut-off value of ≤0.55 is recommended by current European guidelines for the non-invasive estimation of pulmonary hypertension [11]. Given the impact of R-CHD on the RV and the pulmonary vasculature, RV-PA uncoupling is a potential predictor of adverse outcomes also in this population, but strong evidence is currently lacking. The primary aim of our study is to investigate the ability of RV-PA uncoupling to predict the occurrence of adverse cardiovascular events in adult patients with R-CHD.

## 2. Materials and Methods

### 2.1. Study Design and Population

For the present study, all cases of R-CHD evaluated for the first time at our adult CHD (ACHD) outpatient clinic between January 2014 and November 2023 (*n* = 202) were reviewed. R-CHD was defined as either an anatomical defect involving the right cardiac chambers and valves (such as Ebstein anomaly, repaired tetralogy of Fallot [rToF], or repaired congenital pulmonary valve disease), or anomalies leading to right heart volume overload (including atrial septal defect [ASD] and partial anomalous pulmonary venous return). Exclusion criteria were: (i) absence of tricuspid regurgitation (TR), preventing the echocardiographic estimation of pulmonary artery systolic pressure (PASP); (ii) more than mild increase in RV systolic pressure due to any form of right ventricular outflow tract obstruction native/residual pulmonary valve stenosis, to avoid potential misinterpretations of PASP estimation; (iii) Eisenmenger syndrome; (iv) any concomitant left-sided lesion; (v) a follow-up period shorter than one year. Clinical and echocardiographic data at the time of the first visit were retrieved from the digitalized medical records database of our hospital. The severity of the CHD lesion was assessed according to the classification proposed by the latest ESC guidelines [12]. The present study is a purely retrospective study. The data is sourced from the “Prognosis in Adult Patients with Congenital Heart Disease, PRO-ACHD” study and conducted within its framework. It does not include clinical trial results. This clinical trial has been approved by the local Ethics Committee (ID: 4742) and registered on ClinicalTrials.gov (ID: NCT06723704). All patients provided informed consent to participate in the study and for clinical follow-up. The study was conducted in accordance with the principles of the 1975 Declaration of Helsinki.

### 2.2. Echocardiographic Assessment

Comprehensive echocardiographic examinations were performed using commercially available ultrasound systems (Philips Epiq 7, Amsterdam, The Netherlands; GE Vivid E95, GEVingmed, Horten, Norway), by experienced cardiologists specialized in the imaging and care of this population (FG, GI, RL, and MCM) as previously reported [13] and complete echocardiographic data were retrieved from the local electronic patients’ information system. TAPSE was measured by M-mode recordings of the lateral tricuspid annulus, whereas PASP was calculated from the peak velocity of the tricuspid regurgitant jet according to the Bernoulli equation plus the estimated right atrial pressure (according to the diameter and inspiratory collapse of the inferior vena cava) [14]. The ratio between TAPSE and PASP was calculated and adopted as a non-invasive surrogate of RV-PA coupling, using a cut-off value of ≤0.55, based on current recommendations [11].

### 2.3. Study Outcomes

Clinical follow-up data were collected by reviewing the medical records from the last visit at our ACHD outpatient clinic or last hospital admission, or through phone contact. In the latter case, standardized telephone interviews were conducted by trained personnel, systematically assessing hospitalizations, emergency department visits, and major clinical events, including those occurring outside our healthcare system, for which details and discharge documentation were requested. The primary composite endpoint included: all-cause mortality, major supraventricular and ventricular arrhythmias (atrial fibrillation, atrial flutter, sustained supraventricular tachycardia, non-sustained and sustained ventricular tachycardia, ventricular fibrillation), unplanned cardiac hospitalizations and need for surgical or percutaneous (re)-intervention during follow-up. For time-to-event analyses, time zero was defined as the date of the baseline echocardiographic evaluation performed at the first ACHD outpatient visit. Only events occurring after this assessment were considered. The event date corresponded to the documented date of occurrence (first arrhythmic episode, hospital admission date, or procedure date). Although in 6 patients an indication for multidisciplinary discussion to establish the need for intervention was formulated at the first visit, no procedure was deemed urgent nor performed within 90 days from baseline assessment.

### 2.4. Statistical Analysis

Data distribution was assessed according to the Kolmogorov–Smirnov test. Continuous variables were expressed as mean ± standard deviation or as median (interquartile range, IQR). Categorical data were expressed as number (percentage). A comparison between patients with and without RV-PA uncoupling was performed. Continuous variables were compared using an unpaired Student’s *t*-test or Mann–Whitney U test, as appropriate and categorical data were evaluated using the Chi2 test or Fisher’s exact test, as appropriate. A *p*-value < 0.05 was considered statistically significant. Differences between patients who experienced any component of the primary composite endpoint during follow-up were assessed following the same statistical methodology. Receiver operating characteristic curve analysis was performed to assess the ability of TAPSE/PASP to predict the composite primary endpoint and the optimal cut-off value. Univariable Cox proportional hazards regression analysis was performed to identify predictors of the primary composite endpoint. Multivariable Cox regression analysis was performed including all variables significantly associated with outcomes at univariable analysis (*p*  <  0.05). The proportional hazards assumption was assessed using Schoenfeld residuals and was not violated. To address potential redundancy among variables, alternative models were fitted and compared using the Akaike Information Criterion (AIC). To account for potential anatomical heterogeneity, we performed a predefined subgroup analysis comparing patients with rToF versus non-rToF R-CHD. Cox proportional hazards models were fitted separately within each subgroup to evaluate the association between RV–PA uncoupling and the primary composite endpoint. Interaction between rToF status and RV–PA uncoupling was formally tested by including an interaction term in the Cox regression model. A clinically grounded multivariable model was also constructed a priori including age, sex, rToF status, prior intervention, and RV mid-diameter. Cumulative event-free survival rates, stratified by the presence of RV-PA uncoupling, were calculated using the Kaplan–Meier method. A secondary analysis to assess the predictors of the need for (re)-intervention (one of the primary composite endpoint determinants) was also conducted. All analyses were performed using SPSS (SPSS version 26, Inc., Chicago, IL, USA) statistical software.

## 3. Results

### 3.1. Study Population Characteristics

**Figure 1:**
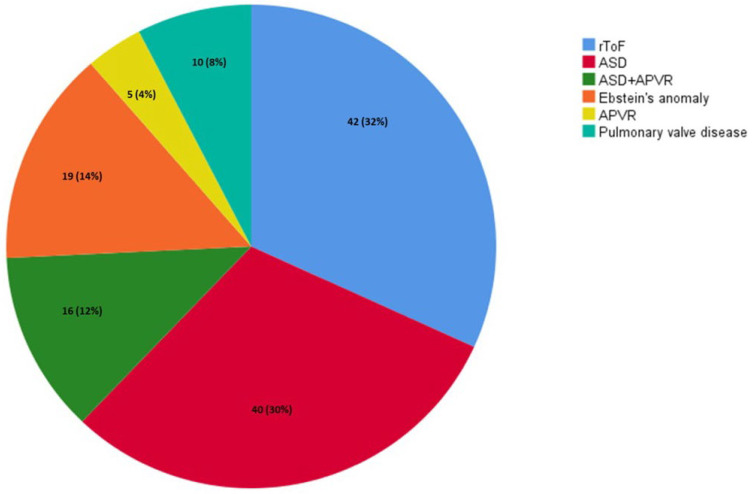
CHD distribution in the study population. Abbreviations: APVR: anomalous pulmonary venous return; ASD: atrial septal defect; CHD: congenital heart disease; rToF: repaired tetralogy of Fallot.

Baseline clinical and echocardiographic characteristics of the overall study population are reported in Table 1. The study cohort consisted of 132 patients [mean age 41.6 ± 15.7 years, 68 males (51.5%)]. The most represented diseases were rToF (42, 32%) and ASD (40, 30%); R-CHD type distribution is illustrated in Figure 1. A total of 56 (42.4%) patients had a mild CHD, while 76 (57.6%) had a moderate CHD. At first clinical evaluation, 13 (9.8%) were in NYHA class ≥ III, 77 (58.3%) had undergone at least one previous surgical/percutaneous intervention, amongst which 26 had received ≥2 operations, and 10 (7.6%) had already a pacemaker/implantable cardioverter-defibrillator. RV and LV systolic functions were within the normal range in most patients (TAPSE 21.3 ± 5.7 mm, LVEF 60.3% ± 6). Mean RV mid-diameter was mildly increased (37.1 ± 11.3 mm), while mean LV end-diastolic volume was within normal limits (84.1 ± 31 mL). Similarly, most patients presented enlarged RA volume [75 mL (IQR 47–99)] and normal LA volume [49 mL (IQR 39–65)]. Moderate or severe TR and pulmonary regurgitation (PR) were detected in 28 (21.2%) and in 21 (15.9%) patients, respectively. The mean TAPSE/PASP value was 0.69 ± 0.29 mm/mmHg. ROC curve analysis identified an optimal TAPSE/PASP cut-off value of 0.56 for predicting adverse events in our cohort (sensitivity 65%, specificity 92%), closely approximating the prespecified threshold of 0.55. The area under the curve was 0.75 (95% CI 0.67–0.84; *p* < 0.001), indicating good discriminative ability (Figure S2).

### 3.2. Characteristics of Patients with RV-PA Uncoupling

**Figure 2:**
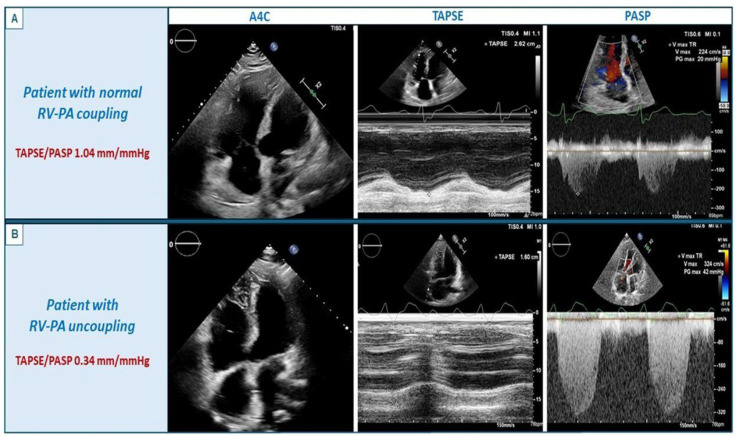
Panel (**A**): Echocardiographic images of a patient with repaired tetralogy of Fallot and normal RV-PA coupling (TAPSE/PASP 0.93). Panel (**B**): Echocardiographic images of a patient with repaired tetralogy of Fallot and RV-PA uncoupling (TAPSE/PASP 0.31). Abbreviations: A4C: apical four chamber view; PASP: pulmonary artery systolic pressure; RV-PA: right ventricle–pulmonary artery; TAPSE: tricuspid annular plane systolic excursion.

A total of 48 (36.4%) patients had RV-PA uncoupling at their first evaluation. The differences between patients with RV-PA uncoupling and those without are depicted in Table 1. Patients with RV-PA uncoupling were more commonly men [62.5% vs. 45.2%, *p* = 0.04] and they were older (46 ± 15 vs. 39 ± 15 years, *p* = 0.02). Moreover, in comparison to their counterparties, patients with RV-PA uncoupling had undergone more frequently multiple previous surgical/percutaneous interventions [37.5% vs. 9.5%, *p* = 0.001] and pacemaker/implantable cardioverter-defibrillator implantation (14.6% vs. 3.6%, *p* = 0.02) and they presented more frequently with NYHA class ≥ III (16.7% vs. 6%, *p* = 0.047). RV-PA uncoupling was also significantly associated with larger RV mid-diameter (40.5 ± 13.1 mm vs. 35.1 ± 9.7 mm, *p* = 0.013), larger RA volume [90 mL (IQR 57.5–135.5) vs. 68 (IQR 45.7–84.5), *p* = 0.009], lower LVEF (58% ± 6.9 vs. 61.7% ± 4.9, *p* = 0.001) and larger LA volume [54 mL (IQR 39.3–80.3) vs. 46 mL (IQR 39–58.2), *p* = 0.04]. Patients with RV-PA uncoupling had both lower TAPSE (16.9 ± 4.1 mm vs. 23.5 ± 5 mm, *p* < 0.001) and higher PASP (41.7 ± 13.1 mmHg vs. 29.5 ± 7.6 mmHg, *p* < 0.001). Figure 2 illustrates echocardiographic images of two rTOF patients, one with RV-PA uncoupling and one without.

### 3.3. Prognostic Value of RV-PA Uncoupling

Over a median follow-up time of 40.3 months (IQR 17.1–77.7), the composite study endpoint occurred in a total of 71 patients (53.8%), of which 29 experienced more than one adverse event. Table S1 summarizes the event rates of the determinants of the composite endpoint, among which need for (re)-intervention was the most frequent (56, 42.6%). Patients who experienced the primary composite endpoint were older at their first visit to our clinic (*p* = 0.001) and at time of both their first and their last cardiac intervention (*p* = 0.022 and 0.042, respectively). They were also more likely to present RV-PA uncoupling (*p* < 0.001), lower left LVEF (*p* = 0.003), lower TAPSE (*p* = 0.005), higher PASP (*p* < 0.001), larger RV mid-diameter (*p* = 0.001), and higher right atrium volume (*p* = 0.003) (Table 2).

At univariate Cox regression analysis, all these variables were significantly associated with the occurrence of the composite endpoint, except for age at first cardiac intervention and RA volume. (Table 3). Multiple multivariable models were fitted, each including alternatively RV-PA uncoupling (Model 1), TAPSE/PASP ratio (Model 2), and TAPSE and PASP together to avoid redundancy (Table 3). Age was found to be an independent predictor of events in all models. RV-PA uncoupling (HR 4.478, 95% CI [2.573–7.792]; *p* < 0.001), TAPSE/PASP ratio (HR 0.217, 95% CI [0.074–0.639]; *p* = 0.006), and PASP (HR 1.038, 95% CI [1.013–1.063]; *p* = 0.003) were confirmed as independent predictors of events in their respective models (Table 3). The three Cox proportional hazards models were compared using the AIC, with Model 1 (AIC = 523.32) demonstrating a better balance between goodness of fit and model complexity than Model 2 (AIC = 531.72) and Model 3 (AIC = 531.86).

In rToF patients (*n* = 42), RV–PA uncoupling was associated with a significantly increased risk of the primary composite endpoint (HR 3.63, 95% CI 1.44–9.17; *p* = 0.006). In non-rToF patients (*n* = 90), the association remained significant and of similar magnitude (HR 4.38, 95% CI 2.42–7.93; *p* < 0.001). Formal interaction testing did not demonstrate a significant interaction between rToF status and RV–PA uncoupling (*p* for interaction = 0.63), suggesting consistent prognostic value across anatomical substrates (Table S2). Moreover, a clinically grounded multivariable Cox model was constructed including age, sex, rToF status, prior cardiac intervention, and RV mid-diameter (Table S3). In this model, RV–PA uncoupling remained independently associated with the primary composite endpoint (adjusted HR 5.354, 95% CI 2.995–9.573; *p* < 0.001). Increasing age was modestly associated with adverse outcomes (HR 1.017, 95% CI 1.000–1.033; *p* = 0.046), whereas sex, rToF status, and RV mid-diameter were not independently associated with events. Interestingly, prior cardiac intervention was inversely associated with the primary endpoint (HR 0.316, 95% CI 0.166–0.604; *p* < 0.001) (Table S3). The magnitude and direction of the association between RV–PA uncoupling and outcome were consistent with those observed in models derived from univariable screening.

**Figure 3:**
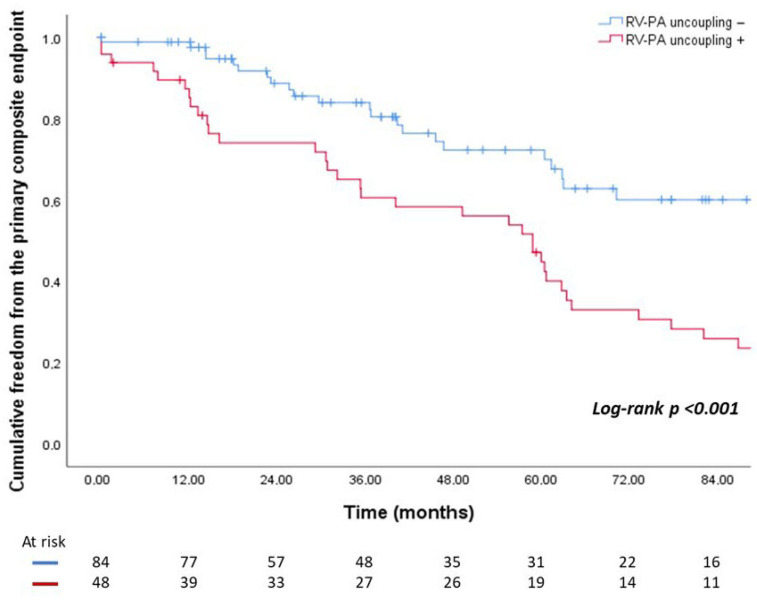
Kaplan–Meier survival analysis assessing the cumulative freedom from the primary composite endpoint in patients with RV-PA uncoupling and in those without. Abbreviations: RV-PA: right ventricle–pulmonary artery.

As shown in Figure 3, patients with RV-PA uncoupling presented significantly higher event rates in comparison with their counterparties at 7-years follow-up (74.1% vs. 41.2%, log-rank test *p* < 0.001).

### 3.4. Predictors of Need for (Re)-Intervention

A secondary analysis was performed to assess the predictors of the single component of the study endpoint, the need for (re)-intervention. A total of 56 (42%) needed surgical/percutaneous operation during follow-up. Patients referred to intervention were more likely to have larger RV mid-diameter (41.4 ± 12.8 mm vs. 33.9 ± 5.2, *p* < 0.001), lower TAPSE/PASP (0.61 ± 0.3 mm/mmHg vs. 0.74 ± 0.27 mm/mmHg, *p* = 0.003) and to present RV-PA uncoupling [32 (57%) vs. 16 (21.1%), *p* < 0.001)] (Table S4). At univariate Cox regression analysis, all these parameters and age at first visit resulted to be predictive of the study endpoint (Table S5). At Cox regression multivariate analysis, only RV-PA uncoupling and larger RV mid-diameter resulted to be independent predictors of need for intervention (HR 2.614, 95% CI [1.488–4.591], *p* = 0.001 and HR 1.025, 95% CI [1.003–1.046], *p* = 0.025, respectively) (Table S5). At survival analysis, RV-PA uncoupling was associated with significantly higher rates of need for intervention (64.5% vs. 37.3%, log rank *p* = 0.007) (Figure S3).

## 4. Discussion

In adult patients with right-sided congenital heart disease (R-CHD), impaired right ventricular to pulmonary artery (RV-PA) coupling—assessed by the TAPSE/PASP ratio—is independently associated with a higher risk of major cardiovascular events, including the need for surgical or percutaneous (re)-intervention. These findings highlight the clinical relevance of RV–PA coupling as a patient-specific functional marker, capable of capturing individual risk beyond anatomical classification alone. Moreover, our results reinforce the long-term adverse impact of chronic pressure and/or volume overload on the right heart [15,16]. Initially, the RV undergoes compensatory (homometric) adaptations to preserve forward flow and protect the pulmonary circulation. Over time, however, sustained wall stress and rising afterload lead to maladaptive (heterometric) remodeling, characterized by progressive ventricular dilation, impaired contractility, and eventual clinical decompensation [16].

In R-CHD, this maladaptation may develop more gradually than in acquired diseases due to chronic exposure to abnormal hemodynamics and the greater adaptive capacity of the congenital heart. Moreover, patients with ACHD often experience a blunted perception of symptoms, delaying recognition of functional decline [1]. This underscores the need for early, objective and individualized markers of RV dysfunction. Importantly, RV-PA coupling has been shown to deteriorate even before overt RV systolic dysfunction in several clinical settings, including CHD [17,18]. Previous studies have shown that RV-PA uncoupling correlates with exercise intolerance and invasive hemodynamics across various ACHD subgroups, including those with pulmonary hypertension [19], pulmonary regurgitation [20], tetralogy of Fallot (ToF) [21,22], and atrial septal defects (ASDs) [18,23,24]. Our study extends these observations by providing long-term prognostic evidence, demonstrating that TAPSE/PASP predicts adverse events independently of underlying lesion severity. This suggests that RV-PA coupling reflects the cumulative hemodynamic burden and functional reserve at the individual patient level, rather than anatomical complexity alone. Notably, TAPSE/PASP was also able to predict the need for (re)-intervention that represents a key clinical challenge and a major source of morbidity in adulthood in this growing population. Current management strategies often rely on expert judgment rather than standardized criteria, and the available evidence remains limited. In this context, simple, non-invasive, and widely accessible parameters such as the TAPSE/PASP ratio may play a pivotal role in guiding personalized surveillance strategies and optimizing the timing of surgical or percutaneous interventions. Overall, given the marked heterogeneity of ACHD and the unique physiology of right-sided lesions, risk assessment requires a multiparametric and personalized approach. The TAPSE/PASP ratio offers a practical, reproducible, and low-cost tool that can support clinicians in identifying high-risk patients and tailoring follow-up intensity and therapeutic decisions accordingly.

### Limitations

We acknowledge some limitations of our study. First, the retrospective design and the relatively small sample size of patients enrolled at a single center may have influenced the results of our analysis. However, most published studies on this topic within the CHD population have even smaller sample sizes or surrogate endpoints. A selection bias cannot be excluded, since a consistent proportion of patients screened was then excluded from the study. Even though the exclusion of patients without measurable TR did not introduce a clinically meaningful imbalance in baseline characteristics (Table S6), the potential for residual selection bias cannot be entirely excluded. In addition, TAPSE was used as the only parameter to assess RV systolic function due to the substantial amount of missing data for complementary RV functional indices, and this may not be the most appropriate choice for rToF. However, TAPSE is by far the most used parameter used for the echocardiographic assessment of RV function. Finally, although we used a composite endpoint to increase statistical power, the majority of events were driven by re-interventions, and this should be considered when interpreting the magnitude and clinical implications of the association.

## 5. Conclusions

RV–PA uncoupling, assessed by the TAPSE/PASP ratio, is an independent predictor of adverse outcomes in adults with R-CHD and represents a valuable tool for personalized risk stratification and individualized clinical decision-making.

## Figures and Tables

**Table 1:** Clinical and echocardiographic characteristics of the overall study population and stratified for RV-PA uncoupling.

Variables	Total Cohort (*n* = 132)	RV-PA Uncoupling + (*n* = 48)	RV-PA Uncoupling − (*n* = 84)	*p*-Value
Age (years)	41.6 ± 15.7	45.5 ± 15.5	39.4 ± 15.5	**0.02**
Males (*n*, %)	68 (51.5)	30 (62.5)	38 (45.2)	**0.04**
Complexity of CHD (*n*, %)				
Mild	56 (42.4)	17 (35.4)	39 (46.4)	0.218
Moderate	76 (57.6)	31 (64.6)	45 (53.6)	
Surgery in pediatric age (*n*, %)				
None	55 (41.7)	15 (31.3)	40 (47.6)	0.09
Corrective surgery	69 (52.3)	28 (58.3)	41 (48.8)	
Palliative surgery	8 (6)	5 (10.4)	3 (3.6)	
Number of previous interventions (*n*, %)				
None	55 (41.7)	15 (31.3)	40 (47.6)	
One	51 (38.6)	15 (31.3)	36 (42.9)	**0.001**
Multiple	26 (19.7)	18 (37.4)	8 (9.5)	
Age at first surgery (years)	8.9 ± 13	10.2 ± 15.6	7.9 ± 11	0.36
Age at last surgery (years)	14.4 ± 16.1	20.3 ± 18.8	10.2 ± 12.5	**0.008**
Previous implantation of PM/ICD (*n*, %)	10 (7.6)	7 (14.6)	3 (3.6)	**0.02**
NYHA class ≥ III (*n*, %)	13 (9.8)	8 (16.7)	5 (6)	**0.047**
LVEF (%)	60.3 ± 6	58 ± 6.9	61.7 ± 4.9	**0.001**
LVEDV (mL)	84.1 ± 31	85 ± 28	83.7 ± 32.8	0.52
LVESV (mL)	33.3 ± 15	35 ± 13.8	32.3 ± 15.6	0.13
LAV (mL)	49 (39–65)	54 (39.3–80.3)	46 (39–58.2)	**0.04**
TAPSE (mm)	21.3 ± 5.7	16.9 ± 4.1	23.5 ± 5	**<0.001**
RV mid-diameter (mm)	37.1 ± 11.3	40.5 ± 13.1	35.1 ± 9.7	**0.013**
RAV (mL)	75 (47–99)	90.5 (57.5–135.5)	68 (45.7–84.5)	**0.009**
PASP (mmHg)	34 ± 11.5	41.7 ± 13.1	29.5 ± 7.6	**<0.001**
TAPSE/PASP (mm/mmHg)	0.69 ± 0.29	0.42 ± 0.1	0.8 ± 0.25	**<0.001**
≥Moderate right-sided valve disease (*n*, %)				
Tricuspid regurgitation	28 (21.2)	11 (22.9)	17 (20.2)	0.71
Pulmonary regurgitation	21 (15.9)	11 (22.9)	10 (11.9)	0.12

Values are expressed as mean ± SD, median (IQR) or *n* (%). Bold values indicate statistical significance at the *p* < 0.05 level. Abbreviations: LAV: left atrial volume; LVEDV: left ventricular end-diastolic volume; LVEF: left ventricular ejection fraction; LVESV: left ventricular end-systolic volume; NYHA: New York Heart Association; PASP: pulmonary artery systolic pressure; PM/ICD: pacemaker/implantable cardioverter device; RAV: right atrial volume; RV: right ventricle; RV-PA: right ventricle–pulmonary artery; TAPSE: tricuspid annular plane systolic excursion.

**Table 2:** Comparison between patients who experienced the primary composite endpoint and those who did not.

Variables	Primary Composite Endpoint (*n* = 71)	No Primary Composite Endpoint (*n* = 61)	*p*-Value
Age (years)	45.3 ± 15.5	37.2 ± 14.9	**0.001**
Males (*n*, %)	42 (59.1)	26 (42.6)	0.06
Complexity of CHD (*n*, %)			
Mild	28 (39.4)	28 (45.9)	0.454
Moderate	43 (60.6)	33 (54.1)	
Surgery in pediatric age (*n*, %)			
None	31 (43.7)	24 (39.3)	0.72
Corrective surgery	35 (49.3)	34 (55.7)	
Palliative surgery	5 (7)	3 (5)	
Number of previous interventions (*n*, %)			
None	31 (43.7)	24 (39.3)	
One	24 (33.8)	27 (44.3)	0.35
Multiple	16 (22.5)	10 (16.4)	
Age at first surgery (years)	11.2 ± 14.8	6.7 ± 10.9	**0.02**
Age at last surgery (years)	18 ± 18.1	11.1 ± 13.3	**0.04**
Previous implantation of PM/ICD (*n*, %)	7 (9.9)	3 (4.9)	0.31
NYHA class ≥ III	9 (12.7)	4 (6.6)	0.24
LVEF (%)	59 ± 6.3	61.9 ± 5.3	**0.003**
LVEDV (mL)	83.8 ± 29.8	84.5 ± 32.7	0.99
LVESV (mL)	33.5 ± 12.8	33.1 ± 17.3	0.45
LAV (mL)	54 (39–73.5)	44 (39–56)	0.11
TAPSE (mm)	19.9 ± 5.8	22.6 ± 5.2	**0.005**
RV mid-diameter (mm)	40 ± 12.3	33.7 ± 8.9	**0.001**
RAV (mL)	85.5 (55.5–121)	60 (37–81.5)	**0.003**
PASP (mmHg)	37 ± 12.5	30.3 ± 9.2	**<0.001**
TAPSE/PASP (mm/mmHg)	0.59 ± 0.28	0.79 ± 0.26	**<0.001**
RV-PA uncoupling (*n*, %)	44 (61.9)	4 (6.6)	**<0.001**
≥Moderate right-sided valve disease (*n*, %)			
Tricuspid regurgitation	18 (25.3)	10 (16.4)	0.21
Pulmonary regurgitation	14 (19.7)	7 (11.5)	0.09

Values are expressed as mean ± SD, median (IQR) or *n* (%). Bold values indicate statistical significance at the *p* < 0.05 level. Abbreviations: LAV: left atrial volume; LVEDV: left ventricular end-diastolic volume; LVEF: left ventricular ejection fraction; LVESV: left ventricular end-systolic volume; NYHA: New York Heart Association; PASP: pulmonary artery systolic pressure; PM/ICD: pacemaker/implantable cardioverter device; RAV: right atrial volume; RV: right ventricle; RV-PA: right ventricle–pulmonary artery; TAPSE: tricuspid annular plane systolic excursion.

**Table 3:** Univariable and multivariable cox regression analysis.

Univariable Analysis			Multivariable Analysis					
			Model 1		Model 2		Model 3	
Variables	Crude HR (95% CI)	*p*-Value	Adjusted HR (95% CI)	*p*-Value	Adjusted HR (95% CI)	*p*-Value	Adjusted HR (95% CI)	*p*-Value
Age (years)	1.031 (1.015–1.048)	**<0.001**	1.018 (1.000–1.035)	**0.004**	1.023 (1.012–1.034)	**0.021**	1.020 (1.002–1.037)	**0.025**
Sex	0.989 (0.608–1.607)	0.964	-	-	-	-	-	-
Age at first surgery (years)	1.021 (0.998–1.045)	0.089	-	-	-	-	-	-
Age at last surgery (years)	1.024 (1.005–1.043)	**0.012**	-	-	-	-	-	-
LVEF (%)	0.948 (0.911–0.986)	**0.007**	0.981 (0.942–1.021)	0.349	0.972 (0.931–1.016)	0.208	0.976 (0.932–1.023)	0.317
TAPSE (mm)	0.952 (0.912–0.995)	**0.027**	-	-	-	-	0.971 (0.924–1.020)	0.235
RV mid-diameter (mm)	1.031 (1.012–1.051)	**0.002**	1.013 (0.991–1.035)	0.251	1.012 (0.990–1.034)	0.288	1.007 (0.984–1.030)	0.540
RAV (mL)	1.001 (1.000–1.003)	0.121	-	-	-	-	-	-
PASP (mmHg)	1.053 (1.032–1.075)	**<0.001**	-	-	-	-	1.038 (1.013–1.063)	**0.003**
TAPSE/PASP (mm/mmHg)	0.136 (0.051–0.361)	**<0.001**	-	-	0.217 (0.074–0.639)	**0.006**	-	-
RV-PA uncoupling	2.530 (1.55–4.118)	**<0.001**	4.478 (2.573–7.792)	**<0.001**	-	-	-	-

Bold values indicate statistical significance at the *p* < 0.05 level. Abbreviations: HR: hazard ratio; LVEF: left ventricular ejection fraction; PASP: pulmonary artery systolic pressure; RAV: right atrial volume; RV-PA: right ventricle–pulmonary artery; TAPSE: tricuspid annular plane systolic excursion.

## Data Availability

The original contributions presented in this study are included in the article/Supplementary Materials. Further inquiries can be directed to the corresponding author.

## Supplementary Materials

The following supporting information can be downloaded at https://www.mdpi.com/article/10.3390/jpm16030164/s1, Table S1: Distribution of the single components of the study endpoint in the overall population; Table S2: Association between RV–PA uncoupling and primary composite endpoint according to rToF status; Table S3: Multivariable clinical model for the composite endpoint occurrence; Table S4: Comparison between patients who needed (re)-intervention and those who did not; Table S5: Univariable and multivariable Cox regression analysis for need for (re)-intervention; Table S6: Baseline characteristics of the study population vs patients excluded for PASP estimation not feasible; Figure S1: Study flow-chart; Figure S2: Receiver operating characteristic curve analysis assessing the ability of TAPSE/PASP to predict adverse events; Figure S3: Kaplan–Meier analysis assessing the cumulative freedom from need for (re)-intervention in patients with RV-PA uncoupling and in those without.
