# Supplementary material for: Prevalence and Prognostic Value of Right Ventricular–Pulmonary Artery Uncoupling in Adults with Right-Sided Congenital Heart Disease
Source: J Pers Med. 2026 Mar 16;16(3):164. doi: 10.3390/jpm16030164 (PMC13028460; doi:10.3390/jpm16030164)
Supplement: Supplementary file 1 [file jpm-16-00164-s001.zip › jpm-4162337-supplementary.pdf]

Supplementary material

Table S1. Distribution of the single components of the study endpoint in the overall population.

Outcome	Total population (n=132)
All-cause death (n,%)	2 (0.02)
Major supraventricular arrhythmias (n,%)	22 (16.6)
Major ventricular arrhythmias (n,%)	7 (5.3)
Unplanned cardiac hospitalization (n,%)	23 (17.4)
Need for (re)-intervention (n,%)	56 (42.4)

Table S2. Association Between RV–PA Uncoupling and Primary Composite Endpoint According to rToF Status.

Subgroup	N	Events	HR	95% CI	*p*-value
rToF	42	25	3.63	1.44–9.17	0.006
Non-rToF	90	46	4.38	2.42–7.93	<0.001
Interaction *p*-value	—	—	—	—	0.63

Abbreviations: r-TOF: repaired Tetralogy of Fallot.

Table S3. Multivariable clinical model for the composite endpoint occurrence.

	Multivariable analysis	
Variables	Adjusted HR (95% CI)	*p-value*
Age (years)	1.017 (1.000-1.033)	**0.046**
Sex	0.867 (0.509-1.478)	0.601
r-TOF	0.855 (0.498-1.785)	0.855
Previous intervention	0.316 (0.166-0.604)	**<0.001**
RV mid-diameter (mm)	1.0.15 (0.994-1.035)	0.162
RV-PA uncoupling	5.354 (2.995-9.573)	**<0.001**

Bold values indicate statistical significance at the p <0.05 level. *Abbreviations:* r-TOF: repaired Tetralogy of Fallot; RV: right ventricle; RV-PA: right ventricle-pulmonary artery; TAPSE/PASP: tricuspid annular plane systolic excursion/pulmonary artery systolic pressure.

Table S4. Comparison between patients who needed (re)-intervention and those who did not.

Variables	Need for (re)-intervention (*n* = 56)	No need for (re)-intervention (*n* = 76)	*p-value*
Age (years)	43.9 ± 15.5	39.8 ± 15.7	0.097
Males (n,%)	32 (57)	36 (47)	0.22
Complexity of CHD (n,%)			
Mild	25 (45)	31 (41)	0.656
Moderate	31 (55)	45 (59)	
Surgery in pediatric age (n,%)			
None	27 (48)	28 (37)	0.321
Corrective surgery	25 (38)	44 (52)	
Palliative surgery	4 (14)	4 (11)	
Number of previous interventions (n,%)			
None	27 (48)	28 (37)	
One	16 (29)	35 (46)	0.09
Multiple	13 (23)	13 (17)	
Age at first surgery (years)	4.5 (1.8-15.3)	3 (0-8)	0.11

Aget at last surgery (years)	11 (3-21.3)	5.5 (1.3-24)	0.37
Previous implantation of PM/ICD (n,%)	3 (5)	7 (9)	0.39
NYHA class ≥ III	4 (7)	9 (12)	0.37
LVEF (%)	59.8 ± 5.4	60.6 ± 6.5	0.18
LVEDV (ml)	79.5 ± 26	87.6 ± 34	0.23
LVESV (ml)	31 ± 9.2	35 ± 17.9	0.51
LAV (ml)	49.5 (38.3-68)	49 (39-61.9)	0.98
TAPSE (mm)	20.2 ± 6.2	22.1 ± 5.2	0.054
RV mid-diameter (mm)	41.4 ± 12.8	33.9 ± 8.9	**<0.001**
RAV (ml)	85 (57.5-102.5)	70.5 (42.5-89.3)	0.07
PASP (mmHg)	36.4 ± 12.7	32.3 ± 10.3	0.051
TAPSE/PASP (mm/mmHg)	0.61 ± 0.29	22 ± 5	**0.003**
RV-PA uncoupling (n,%)	32 (57)	16 (21)	**<0.001**
≥ Moderate right-sided valve disease (n,%)			
Tricuspid regurgitation	12 (21)	16 (21)	0.96
Pulmonary regurgitation	12 (21)	9 (12)	0.12

Values are expressed ad mean ± SD, median (IQR) or n (%). Bold values indicate statistical significance at the p <0.05 level. *Abbreviations:* CHD: congenital heart disease; LAV: left atrial volume; LVEDV: left ventricular end-diastolic volume; LVEF: left ventricular ejection fraction; LVESV: left ventricular end-systolic volume; NYHA: New York Heart Association; PASP: pulmonary artery systolic pressure; PM/ICD: pacemaker/implantable cardioverter device; RAV: right atrial volume; RV: right ventricle; RV-PA: right ventricle-pulmonary artery; TAPSE: tricuspid annular plane systolic excursion.

Table S5. Univariable and Multivariable Cox regression analysis for need for (re)-intervention.

	**Univariable analysis**		**Multivariable analysis**	
Variables	Crude HR (95% CI)	*p-value*	Adjusted HR (95% CI)	*p-value*
Age (years)	1.023 (1.006-1.041)	**0.009**	1.011 (0.992-1.030)	0.262
Sex	0.866 (0.506-1.483)	0.601	-	-
Complexity of CHD	0.773 (0.450-1.327)	0.350	-	-
NYHA class ≥ III	1.108 (0.400-3.072)	0.844	-	**-**
LVEF (%)	0.977 (0.933-1.024)	0.331	-	-
RV mid-diameter (mm)	1.038 (1.017-1.058)	**<0.001**	1.025 (1.003-1.046)	**0.025**
TAPSE/PASP (mm/mmHg)	0.200 (0.071-0.567)	**0.002**	-	-
RV-PA uncoupling	3.162 (1.850-5.405)	**<0.001**	2.614 (1.488-4.591)	**0.001**

Bold values indicate statistical significance at the p <0.05 level. *Abbreviations:* CHD: congenital heart disease; HR: hazard ratio; LVEF: left ventricular ejection fraction; NYHA: New York Heart Association; RV: right ventricle; RV-PA: right ventricle-pulmonary artery; TAPSE/PASP: tricuspid annular plane systolic excursion/pulmonary artery systolic pressure.

Table S6. Baseline characteristics of the study population vs patients excluded for PASP estimation not feasible.

**Variables**	**Study population (*n* = 132)**	**Patients excluded for PASP estimation not feasible (*n* = 26)**	***p*-value**
Age (years)	41.6 ± 15.7	40.7 ± 14.6	0.78
Males (n, %)	68 (51.5)	14 (53.8)	0.828
Moderate CHD (n, %)	76 (57.6)	13 (50)	0.477
NYHA class ≥ III (n,%)	13 (9.8)	2 (7.7)	0.732
LVEF (%)	60.3 ± 6	62.3 ± 5	0.079
LVEDV (ml)	84.1 ± 31	85.6 ± 36	0.84
LVESV (ml)	33.3 ± 15	31.4 ± 13	0.51
LAV (ml)	49 (39–64.8)	43 (34–55)	0.26
RAV (ml)	75 (47–98.5)	69 (46.8–85.4)	0.41
RV mid-diameter (mm)	37.1 ± 11.3	34.3 ± 10.8	0.24

TAPSE (mm)	21.3 ± 5.7	22.6 ± 4.6	0.21

Values are expressed ad mean ± SD, median (IQR) or n (%). *Abbreviations:* CHD: congenital heart disease; LAV: left atrial volume; LVEDV: left ventricular end-diastolic volume; LVEF: left ventricular ejection fraction; LVESV: left ventricular end-systolic volume; NYHA: New York Heart Association; PASP: pulmonary artery systolic pressure; RAV: right atrial volume; RV: right ventricle; TAPSE: tricuspid annular plane systolic excursion.

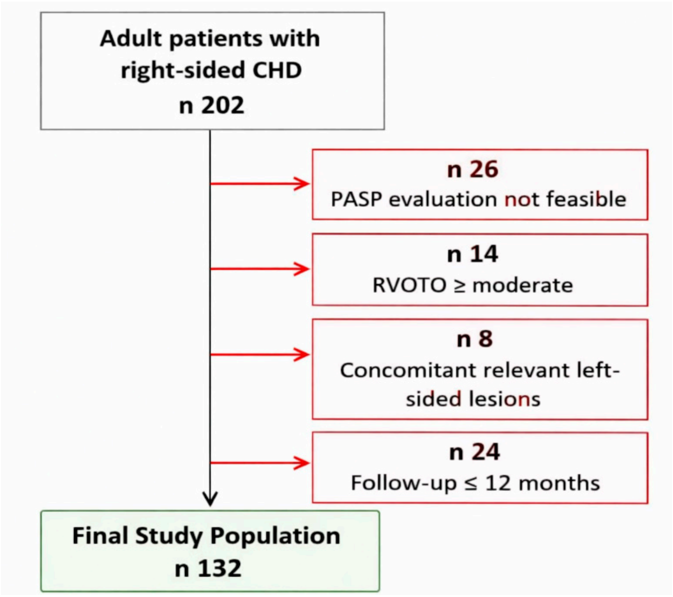

Figure S1. Study flow-chart. *Abbreviations:* CHD: congenital heart disease.

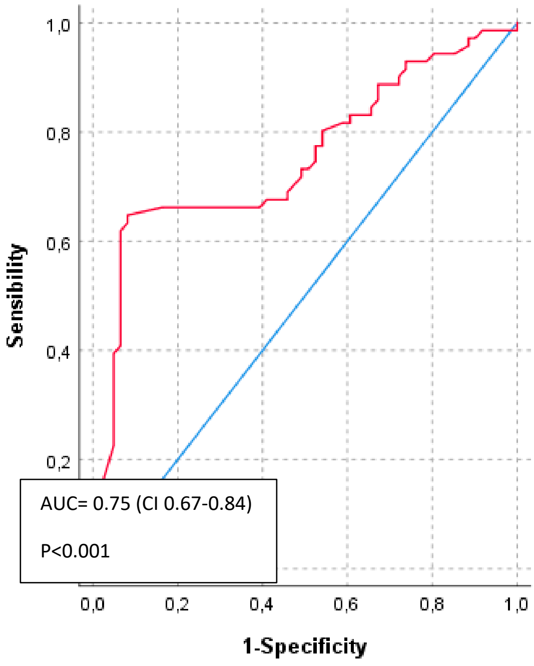

Figure S2. Receiver Operating Characteristic Curve analysis assessing the ability of TAPSE/PASP to predict adverse events. *Abbreviations:* AUC: area under the curve.

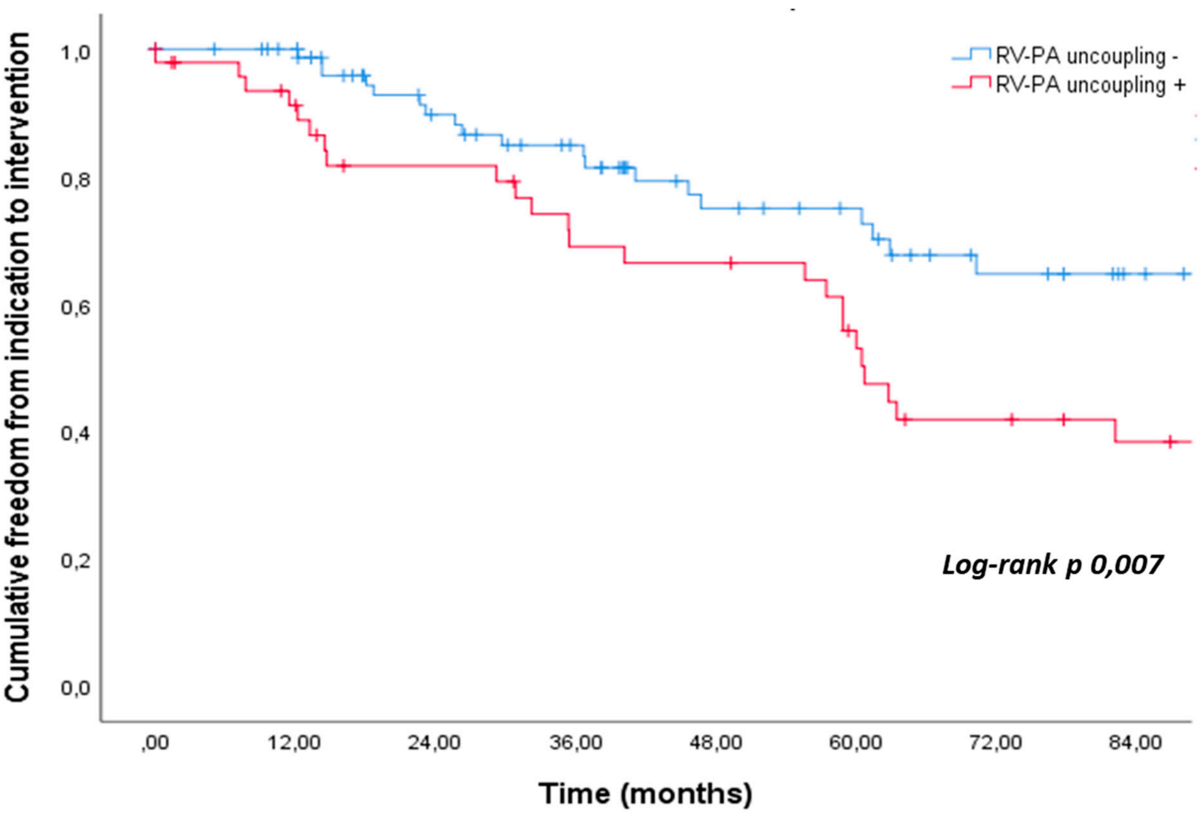

At risk

blue line	84	76	57	48	35	31	22	16
red line	48	39	33	27	26	19	14	11

Figure S3. Kaplan–Meier analysis assessing the cumulative freedom from need for (re)-intervention in patients with RV-PA uncoupling and in those without. *Abbreviations:* RV-PA: right ventricle-pulmonary artery.
